# Disabled women׳s maternal and newborn health care in rural Nepal: A qualitative study

**DOI:** 10.1016/j.midw.2014.03.012

**Published:** 2014-11

**Authors:** Joanna Morrison, Machhindra Basnet, Bharat Budhathoki, Dhruba Adhikari, Kirti Tumbahangphe, Dharma Manandhar, Anthony Costello, Nora Groce

**Affiliations:** a Institute for Global Health, University College London, 30 Guildford Street, London WC1N 1EH, UK; b MIRA, PO Box 921, Thapathali, Kathmandu, Nepal; c Leonard Cheshire Disability and Inclusive Development Centre, Department of Epidemiology and Public Health, University College London, 1-19 Torrington Place, London WC1E 6BT, UK

**Keywords:** Disability, Neonatal, Equity, Quality, Access, Respectful care

## Abstract

**Objective:**

there is little evidence about disabled women׳s access to maternal and newborn health services in low-income countries and few studies consult disabled women themselves to understand their experience of care and care seeking. Our study explores disabled women׳s experiences of maternal and newborn care in rural Nepal.

**Design:**

we used a qualitative methodology, using semi-structured interviews.

**Setting:**

rural Makwanpur District of central Nepal.

**Participants:**

we purposively sampled married women with different impairments who had delivered a baby in the past 10 years from different topographical areas of the district. We also interviewed maternal health workers. We compared our findings with a recent qualitative study of non-disabled women in the same district to explore the differences between disabled and non-disabled women.

**Findings:**

married disabled women considered pregnancy and childbirth to be normal and preferred to deliver at home. Issues of quality, cost and lack of family support were as pertinent for disabled women as they were for their non-disabled peers. Health workers felt unprepared to meet the maternal health needs of disabled women.

**Key conclusions and implications for practice:**

integration of disability into existing Skilled Birth Attendant training curricula may improve maternal health care for disabled women. There is a need to monitor progress of interventions that encourage institutional delivery through the use of disaggregated data, to check that disabled women are benefiting equally in efforts to improve access to maternal health care.

## Introduction

Disabled persons are estimated to constitute 15% of the world׳s population and a disproportionate percentage of the world׳s poor (WHO and The World Bank, 2011, Hosseinpoor et al., 2013, Mitra et al., 2013). Their inclusion in development efforts is essential for the attainment of the Millennium Development Goals (MDGs), but tracking progress among disabled people is difficult as disaggregated data are often unavailable (WHO and The World Bank, 2011). Equity analyses of MDG targets for maternal and child health have been useful in highlighting inequalities in access to life saving interventions between rich and poor (Barros et al., 2012), and there is little evidence regarding the access of disabled women to reproductive health services in low income countries. There is also a paucity of research that asks disabled women themselves about their experiences and opinions about the maternal health services they receive.

We present qualitative findings from research to explore disabled women׳s maternal and newborn health care in rural Nepal. Our research partnership of the Institute for Global Health, University College London (UCL), and MIRA Nepal has been testing the impact of community-based interventions on maternal and newborn survival through cluster randomised controlled trials. Working in partnership with the Leonard Cheshire Disability and Inclusive Development Centre at UCL, we have completed mixed methods research to describe severely disabled women׳s maternal and newborn care behaviours using trial surveillance data, and qualitative data. In this paper we present qualitative findings from this research. We discuss our findings in comparison with recent research conducted with women delivering at home and in health institutions without severe disabilities in the same district (Morrison et al., 2014).

### Sexual and reproductive health of disabled women

Women with disabilities have largely been ignored in reproductive health research. They are often thought not to be sexually active, and less likely to marry or to have children than non-disabled women (Kallianes and Rubenfeld, 1997, Anderson and Kitchin, 2000, Milligan and Neufeldt, 2001, Smith and Murray, 2004, WHO and UNFPA, 2009). These beliefs may come from perceptions that disabled women are either ‘passive receivers of help’ (Yousafzai et al., 2004) or ‘patients’ not capable of marriage or giving birth (Grue and Laerum, 2001). However, the literature shows that rates of sexual activity and child birth among disabled women are comparable to those of non-disabled women (WHO and UNFPA, 2009). Although attitudes may be changing in some contexts (Simkhada et al., 2013), stigma and prejudice often prevent access to reproductive health information and services (Yousafzai et al., 2004, Maxwell et al., 2007, Eide et al., 2011).

### Disability in Nepal

The recent national census found that 2% of the population were disabled (513,321) (Central Bureau of Statistics, 2012). Physical and sensory disabilities were the most prevalent. These estimates may be conservative, as other surveys have reported higher incidence of disability (Dhungana, 2006). Our recent survey, which used adapted questions from the Washington Group on Disability Statistics, found that 29% (3930) of married women with children had a mild, moderate or severe impairment (Institute for Global Health et al., 2013). The low figures referred to in the census may also be indicative of differences in how disability is perceived and captured (WHO and The World Bank, 2011, Simkhada et al., 2013). A recent study has also indicated that a national plan of action for persons with disabilities was written in 2006 (Ministry of Women Children and Social Welfare, 2006), and in 2008, Nepal ratified the convention of the rights of persons with disabilities (ESCAP, 2009). The rights of persons with disabilities have also been recognised in the interim constitution (Organisation Development Centre, 2007), yet people with disabilities in Nepal still face significant challenges. The most recent data from 2001 shows that 68% of people with disabilities have no education and 78% have no access to earn a living (UNICEF & National Planning Commission, 2001).

### Status of women in Nepal

Disabled women face additional disadvantage compared with disabled men, as they are discriminated against because of their gender as well as their disability (Dhungana, 2006). Nepal was ranked 113th out of 187 countries in the UN gender inequality index, and 5th among countries of the South Asian Association for Regional Cooperation (UNDP, 2011). Patriarchal and Hindu marriage customs, such as patrilocal residence after marriage, patrilineal descent and inheritance system, and a rigid family hierarchy help maintain women׳s disadvantaged position (Bennett, 2005). Marriages are usually arranged and occur at a relatively early age: the median age at first marriage is 17.8 years, and 16% of women are married before the age of 15 (Ministry of Health and Population, 2011).

### Maternal health

Maternal health indicators for Nepal are improving and it is likely that national programmes of free institutional delivery care and cash transfers have contributed to increases in institutional deliveries over recent years (Powell-Jackson et al., 2010). Cost is not the only barrier to institutional care; quality is often compromised by shortages of equipment and supplies, and a lack of adequately trained health personnel (Government of Nepal, 2010). Nepal׳s short term plan for safe motherhood includes increasing the skills of nurses through a Skilled Birth Attendant training programme (Family Health Division, 2006a, Family Health Division, 2006b), but its long-term plan for the development of midwifery education and a midwife cadre has been slow to progress. A recent feasibility study and pressure from the Midwifery Society of Nepal may help to promote strategic planning for midwifery in Nepal (Bogren et al., 2013). Health service utilisation is affected by the short opening hours of health facilities, geographical barriers, and cultural issues (Pradhan et al., 2010). A fear of bringing shame on the family through discussion of pregnancy provokes concealment and embarrassment among pregnant women, sometimes preventing care seeking when there is a problem (Mesko et al., 2003).

## Methods

### Setting

Makwanpur District is in the central hills of Nepal, and most of its population of 420,477 are rural farmers (Central Bureau of Statistics, 2012). Only 17% of the population live in the urban district centre of Hetauda, where there is a 50-bed government hospital, and three private hospitals. Makwanpur has a mid-range Human Development Index (HDI) of 0.479, and low female literacy (53.9%) (District Development Committee, 2011).

### Data collection

We screened 13,687 married women with at least one child for incidence, type and severity of disability. These women had been participating in maternal and newborn health research from August 2001 to December 2008 in 30 clusters of Makwanpur District. A cluster is a geopolitical unit of the Village Development Committee (VDC). The screening tool was developed and used in the National Disability Survey in Afghanistan (Trani and Bakhshi, 2008). The tool has 35 questions to detect epilepsy, physical, sensory, learning, behavioural, and social function and communication disabilities based on the International Classification of Functioning, Disability and Health (ICF) (WHO, 2001) and Sen׳s capability approach (Sen, 1999). The tool captures severity of disability by asking respondents to rank their status on a four-point Likert scale.

### Sampling

**Fig. 1:**
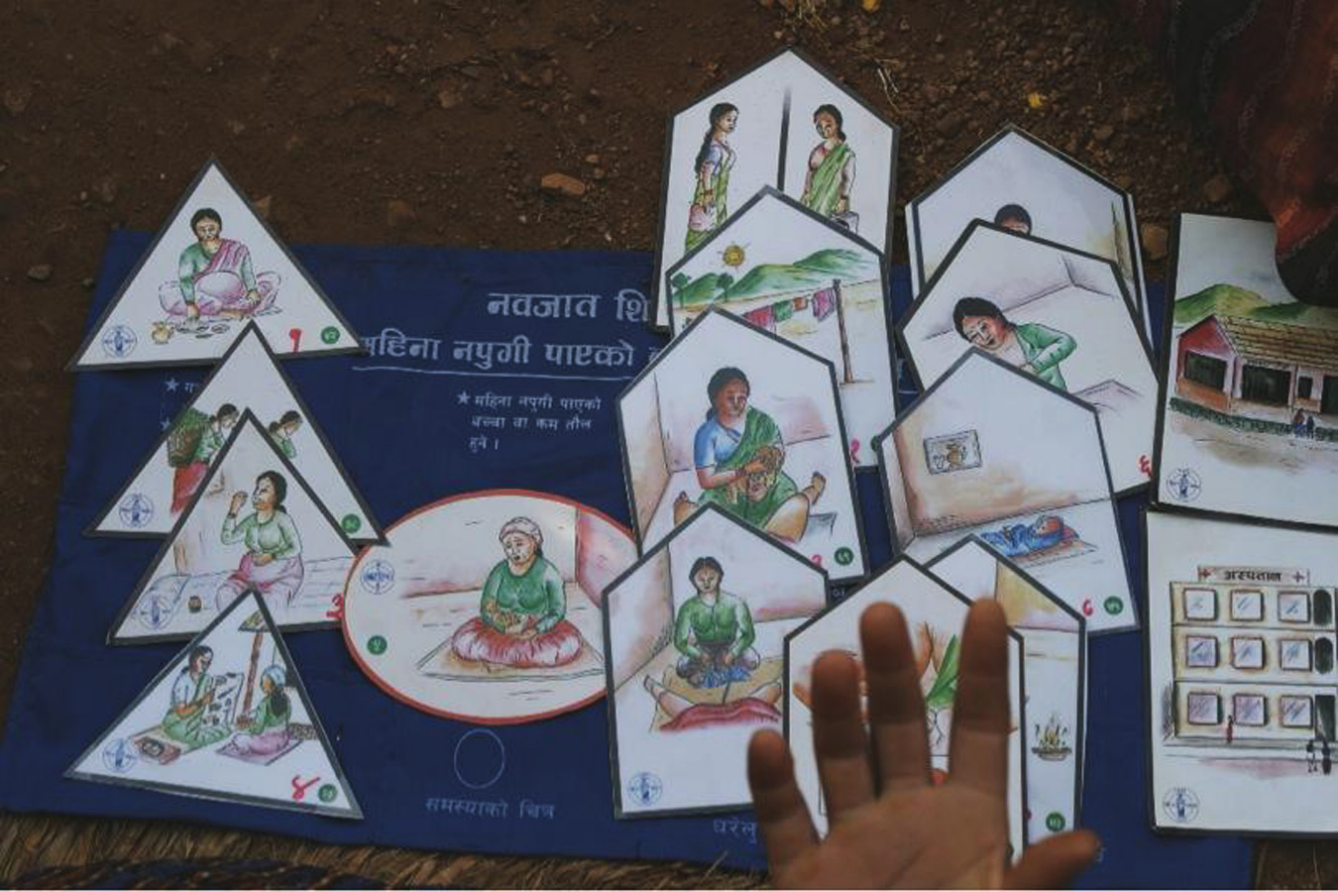
Picture cards used in discussions with disabled women.

We used information from the screening tool to locate disabled women. We purposively sampled four VDCs in the hilly part of the district, and three VDCs in the plains with a high population of severely disabled women. A trained and experienced qualitative researcher (MB) worked with local interviewers to locate and interview 27 severely disabled women and five Auxiliary Nurse Midwives (ANMs). We purposively sampled women with different types of impairment. Women were interviewed about their experience of pregnancy, childbirth and the postpartum period, and we asked them to describe their experiences with the health services. They discussed how their disabled status had affected the maternal care and support they had received at home, in their community, and in the health facility. We used locally developed picture cards during interviews with intellectually impaired and hearing-impaired women (Fig. 1). Open and closed questions were asked, keeping the number of choices minimal for women with intellectual impairments, as they tend to find it difficult to choose from many options. We conducted 10 interviews with the help of family and friends (Table 1). One interview with an intellectually impaired woman was excluded from analysis, as data were contradictory and difficult to interpret. Participants gave verbal consent to participate.

**Table 1:** Characteristics of disabled women.

**Type of impairment**	**Number of women**
Physical	6
Blind	3
Hearing impaired	10
Intellectual	8

**Ethnicity**	
Tamang	15
Brahmin/Chettri	6
Other advantaged group	5
Disadvantaged group	1

**Reported experience of violence and/or abuse**	
Yes	9
No	18

**Household living conditions**	
Extended family	12
Nuclear family	15

**Interviewed with neighbour or family present**	
Yes	12
No	15

**Institutional delivery**	5
**Home delivery**	22
**Total**	27

Data were recorded and transcribed into English. A bilingual researcher checked the quality of two translations. Data were manually analysed by MB and JM (Krueger and Casey, 2000). After familiarisation, we analysed data according to emergent themes. We compared data from health workers and disabled women, and disabled women with different impairment types. We present quotes with identifiers of type of impairment, and topography.

We received ethical approval for this study from the Nepal Health Research Council and University College London Ethics Committee.

## Findings

### Normalcy of pregnancy and home delivery

Pregnancy after marriage is expected in Nepal, and therefore many disabled women were bewildered about how to respond when we asked them how their family felt about their pregnancy. Some reported that no one had said anything in particular about their pregnancy. Others did not explicitly tell their family that they were pregnant. This is not unusual in this context where women are often embarrassed about their pregnancy. For married disabled women, pregnancy was a normal, or inevitable event, and they were as likely as other married women to have children. When asked: ‘How did you feel when you found out you were pregnant?’ a physically disabled woman from the plains responded: ‘How should I feel? I just ate my food and did my work.’

Some women reported that their in-laws were happy about the pregnancy:

I was quite happy. The family members would have been upset if I weren׳t able to conceive a child. (Physically impaired woman, plains)

Home delivery was perceived to be inevitable and best. Women often assumed that they would have no complications, and therefore there would be no need to deliver in a health institution. When asked where she planned to deliver her baby, a hearing impaired woman from the hills told us:

We didn׳t have any plans. All our children have been easily delivered at home.

### Embarrassment

Although pregnancy is considered normal and inevitable for married women, it is also a source of shame and embarrassment. Many disabled women were embarrassed and this often prevented them from telling their in-laws about their pregnancy:

It׳s difficult to tell to parents-in-law isn׳t it? I was frightened to tell my in-laws (Physically impaired woman, plains).

Interestingly, almost all of the participants who reported embarrassment as a barrier to care seeking, had had an antenatal check-up. However, embarrassment tended to affect decisions about place of birth. Women were afraid of bringing shame on their family if they showed their body during institutional delivery. Physically impaired women and hearing impaired women reported feeling more embarrassed than those with other types of impairment, but this appeared unrelated to their disabled status:

I was thinking it would be better if I could normally deliver my baby at home without going to the health facility. We can deliver in privacy at home, but everyone comes and can see our private parts when we go to the health facility (Physically impaired woman, plains).

Health workers found it difficult to provide services to women who were embarrassed:

It was very difficult for me to give service because she was shy and afraid (ANM, hills).

A few respondents mentioned being judged in their community and by health workers for having too many children, which made them embarrassed and reluctant to seek care:

They scolded me when I went for an antenatal check-up saying ‘why are you pregnant again? You already have three children’ (Hearing impaired woman, hills).

This is not unique to disabled women, and non-disabled women have also reported this kind of treatment by health workers in the District.

### Lack of awareness

Health workers felt that disabled women may lack awareness about the services available, or awareness about the importance of seeking health care during pregnancy or childbirth, and this may prevent them from having an institutional delivery. An ANM working in the plains explained:

Lack of awareness among disabled women might be the main problem. They don׳t come to seek service if they are not aware.

### Experience of care

Some women representing the range of all impairment types were satisfied with the antenatal care they had received. Generally, disabled women preferred health workers from the local area, who spoke the local language. Others felt satisfied with the advice and the check-up procedures. One woman stated that the health worker was more attentive to her than with other patients. There were more reports of positive experience among physically impaired women, who were pleased with the advice that they received:

They told me to eat nutritious food. They also gave me iron tablets. Health workers suggested that I should not be alone after my labour starts as I have problems with my hands. They were very good to me (Physically impaired woman hills).

Intellectually impaired women who were satisfied tended not to give details about what aspect of care they liked, and only reported that the services were ‘good’. More hearing impaired women with positive experiences tended to be satisfied with the check-up procedures and medicine in comparison with women who had other types of impairment.

However, experiences reported were not universally positive. Disabled women with a variety of impairment types had experienced some bad treatment. When health workers were unkind and impolite, women were deterred from seeking care again:

(I) went to the health post once and from then onwards (I) didn‘t go because the health workers shouted at (me) (Hearing impaired woman, plains).

To some extent, rude behaviour of health workers was expected, and disabled women did not link the way they were treated with their impairment:

Some of the nurses were rude. Sometimes we have to wait for a very long time for a check up. It׳s normal for them to be rude. I am not only the patient. They have to face many other patients. I never felt bad whenever they were rude to me (Physically impaired woman, plains).

Some disabled women reported being reprimanded for reasons unrelated to their impairment:

One of the nurses shouted at me the first time I went there. I was wearing a blouse and sari and she told me ‘can׳t you come wearing salwar suit (trousers and tunic)?’ (Intellectually impaired woman, hills).

Generally, health workers found it most difficult to provide services to women with hearing and speech impairments, as they could not communicate easily with them. Correspondingly, more hearing impaired women reported negative interactions with health workers:

the health workers tease (me) and get irritated whenever (I) go (to the health facility) (Hearing impaired woman, plains).

There was increased potential for miscommunication between health workers and hearing or speech-impaired women. A hearing impaired woman from the hills gave the example of when she went for a check-up with her daughter-in-law, and she was unclear about who was being referred to the district hospital:

I thought they told my daughter-in-law to go to the district hospital, but actually it was advice for me to go.

Health workers also had difficulties communicating with intellectually impaired women. They found it difficult to explain procedures, and counsel regarding home care. No health workers reported having used gestures or pictures to aid explanation. They relied on accompanying persons to ‘translate’ the messages, through ‘home signs’ and gestures, which they found very unsatisfactory:

It would have been better if I could have made the woman herself understand everything. My advice would have been more effective if she had understood…the person who we told to give the medicines might not give them to her. We cannot completely rely on the third person (ANM, hills).

Health workers reported some difficulties with the infrastructure and equipment in providing care to physically disabled women:

Sometimes it is very difficult for (disabled woman) to lie on the beds which are too high (ANM, plains).

### Social support

In general, physically disabled women reported the most difficulty in performing housework, and caring for themselves and their families. Many disabled women reported receiving support from their family, neighbours and friends during and after childbirth, and more generally. Usually this was in the form of advice about how to care for themselves and their baby and help with housework. Some women also received financial assistance. A blind woman from the plains told us:

Sometimes my neighbours come to cook food for me…(they) help me when problems arise. They lend me money if I need it.

Not all disabled women received support, and some reported being teased or treated differently. A few women felt isolated, and may have expected to be excluded:

We have neighbours but we don׳t care whether they come to visit us or not. No-one talks to me (when I walk around). I walk my own way (Intellectual impaired woman, plains).

The relative of a hearing impaired woman from the plains told us:

She never goes out of the house. She says she feels shy and uncomfortable going outside the house.

There were also data from women with all types of impairment reporting that they cared for their infant themselves because there was no one around to help. This is unusual in this context where family members often take an active role in caring for the newborn. A blind woman from the plains said:

I cut the umbilical cord by myself. There was no-one. I cut the cord where I could see. I cannot see properly.

A hearing impaired woman from the hills also reported being unsupported when she had her first child:

Who would teach me about massage, feeding and bathing? I did everything by myself… no-one was there (Hearing impaired woman, hills).

Although many women were supported and helped by friends and family, one-third of the women we sampled had suffered physical or emotional abuse (Table 1). The friend of a hearing impaired woman from the plains told us:

Her sister-in-law was very rude to her… three days after delivery she hit her and pulled her hair out. Her sister-in-law wanted all her land as she has lots of land in her name. She had more land but her sister-in-law took half of her land by cheating after the death of her parents-in-law.

Few women in our sample reported receiving support from non-governmental organisations, despite the presence of several organisations working on disability issues in the district. We did not ask participants whether they had received the government disability allowance, but one blind woman from the plains told us how difficult it was to get her entitlement:

Other disabled people have been getting 1000 rupees allowance but they only give me 300 Rupees. If you are a person that speaks-up, you get all the allowance, and I don׳t get it all because I don׳t speak-up.

### Economic difficulties

Some participants were very poor, without their own house or land, earning small amounts of money sorting stones, making alcohol, or sewing. This affected their ability to care for themselves and their families:

At night time we didn׳t have a bed to sleep. We were sleeping on the floor. My eight month old son moved towards the fire and burnt his legs (Physically impaired woman, plains).

We wanted to explore to what extent a woman being disabled affected the way that her children were treated. When we asked disabled women, we found that there was no differential treatment of children whose mother was disabled, and poverty affected children more than their mother׳s disability. One blind woman from the plains told us:

There are also some functions that have to be done for our daughters but we cannot perform them as we don׳t have money.

Many disabled women felt that institutional delivery was expensive, and therefore home delivery was preferable:

We need to pay for transport to the health post. After reaching the health post we need money again, for medicine (Intellectually disabled woman, plains).

Some data suggests that poverty was more of a barrier than embarrassment or bad behaviour of health workers, and many women felt it was inevitable that they would deliver at home:

The main reason is the economic problem. We couldn׳t afford the fees to go to hospital. There was no one to support my husband to take me to hospital. My parents don׳t live close-by. My husband wanted to take me to the health facility but I didn׳t want to go as we didn׳t have money for hospital charges (Physically impaired woman, hills).

Some women reported being denied resources by their husbands preventing them from seeking healthcare:

My husband told me not to go to the health facility because he doesn׳t have any money (Intellectually impaired woman, plains).

At the time we administered the screening tool, delivery care was free and incentivised through unconditional cash transfers, but most participants had given birth before this policy change. One physically impaired woman who had delivered more recently was pleased with the free delivery service. She said:

I got everything that I needed. We didn׳t have to use our money during the course of treatment. We got all the medicine free of costs…I am satisfied.

In addition to free delivery care and the incentive, health workers felt that additional incentives for disabled women and their family members would encourage them to come to health institutions. Additional incentives could compensate accompanying family members who were often daily wage labourers.

## Discussion

Married disabled women felt that it was normal and preferable to deliver at home. Many received support from friends and family, but some also reported lack of support, abuse and isolation. Although many were satisfied with the antenatal care they received, few women had had an institutional delivery. They preferred to deliver at home because they were worried about cost, and were embarrassed to show their body. Disabled women reported mixed experiences with health workers. In some cases, an aspect of her disability affected the care she received, whereas other women found the care adequate. Often if they were poorly treated, women felt that their disabled status was not linked to their treatment. When disabled women had had a bad experience with the health services, they were unwilling to use them again. Health workers felt that women were not aware of the benefits of institutional delivery, and often found it difficult to meet their needs.

### Limitations

Women are usually accompanied when they visit health services, and it would have been beneficial to collect data from family members who might have been key support persons in the home or at a health institution. Our qualitative sample was limited to women who had been enroled in previous research and therefore we only sampled disabled married women who had delivered a baby between 2001 and 2008. Most women delivered at home, and therefore we were unable to describe disabled women׳s experience of institutional delivery, and unmarried disabled women׳s access to care. Future studies should consider these issues, as well as disabled women׳s access to free delivery care, incentives, and government social support benefits. This is particularly relevant as disabled people are, on average, poorer than non-disabled people (Hosseinpoor et al., 2013), and a study has shown that awareness of free institutional delivery care was lower among the poor (Powell-Jackson et al., 2010).

### Comparing access among disabled and non-disabled women

For disabled women delivering at home, their disability was rarely their first consideration when making decisions about where to have their baby. When we compared these findings with data from 2012 in the same district from women delivering at home without severe disabilities, we found that the barriers to institutional delivery were similar among disabled and non-disabled women.

The normalcy of home delivery, and the practice of only accessing care when there was a problem, was similar among disabled and non-disabled women delivering at home. Cost was also a universal barrier, with both groups deterred by the cost of transport, and a fear of putting their family into financial difficulty. Many barriers to care seeking were related to women׳s low status in the home and dominance of other family members (Simkhada et al., 2010, Shrestha et al., 2012). Culturally, women are expected not to make a fuss during childbirth, and should not complain or disturb others during delivery (Blanchet, 1984, Huque et al., 1999, Afsana and Rashid, 2001, Head et al., 2011).

Family support to access care was necessary for disabled and non-disabled women. The National Demographic and Health Survey found that only 65% of women, versus 87% of men, said they made decisions regarding their own healthcare (Ministry of Health and Population et al., 2011). Disabled women may occupy lower status in the household than other non-disabled women (Dhungana, 2006), and the relatively large proportion of women reporting abuse in our study may reflect this. We found that some disabled women did not expect family support, and they may have lower expectations about the quality of care they should receive (UNESCAP, 1995). We found that some disabled women expected rude treatment from health workers, and there is evidence to suggest that this may also be the case for non-disabled women who have also reported similar treatment (Pradhan et al., 2010).

### Improved awareness

The recent study of non-disabled women׳s care seeking, found that men, women and health management committee members believed that a lack of awareness about the benefits of institutional delivery prevented care seeking (Morrison et al., 2014). Health workers in this study also believed disabled women and their families suffered from a lack of awareness. Yet non-disabled women themselves were generally aware of the benefits of institutional delivery, and did not feel this was a barrier to care seeking. This may be as a result of a government focus on maternal health over recent years, and our community based interventions since 2001 (Manandhar et al., 2004, Morrison et al., 2010, Morrison, 2011, Morrison et al., 2011). We do not know if newly married disabled women have also benefitted from increased information about maternal health, as our sample of disabled women had delivered babies from 2001 to 2008. The literature suggests that disabled women have less access to reproductive health information (Becker et al., 1997, Anderson and Kitchin, 2000; WHO and UNFPA, 2009), and therefore it is important to understand the extent to which safe motherhood initiatives have reached disabled women.

### Quality of care

Health workers were concerned about the quality of care that they were able to provide to disabled women, and disabled women were unlikely to seek care if they did not like the way they were treated. Yet training health workers on disability is rare. This lack of knowledge about disabled women presents a potential barrier to increasing institutional deliveries and improving quality of care. Mainstreaming disability issues in the national Skilled Birth Attendant training course may help nurses to be better prepared. This, in combination with enhanced interpersonal skills training, and training on respectful care at birth would help to provide better care for all women (Jolivet, 2011). Having a birth companion from the woman׳s household or social network who could help to translate and explain what the health workers are saying may be particularly important for disabled women with communication difficulties or disabled women who have limited ability to understand or recall instructions. In light of this, health workers should focus on building a relationship of trust through multiple contacts with a disabled woman. Understanding what is happening and why is an important component of quality care (Hulton et al., 2000). Increasing health workers׳ confidence in using alternative ways of communicating (gestures, pictures and sign language) may help interactions between health workers and disabled women. In Nepal, the absence of a midwifery cadre is an impediment to providing better care for all women, but disabled women could particularly benefit from the woman-centred care that is midwifery.

Quality issues were also of concern to non-disabled women (Morrison et al., 2014). They criticised the short opening hours and lack of health personnel. During childbirth they had less freedom to move around, and could not give birth in the position of their choice. This lack of flexibility by health workers might affect disabled women particularly. Non-disabled women also reported missing home comforts, such as family support during childbirth, warmth, and access to food and drink. Both disabled and non-disabled women were embarrassed about showing their body during childbirth. If privacy were better maintained, this could lessen embarrassment and positively affect utilisation of services by all women.

### Impairment in context

Whether a condition becomes a disability may be context dependent (UN, 2006). A recent study suggests that Nepali women may not perceive disabilities in the same way that the WHO defines (Simkhada et al., 2013). Religious and social personhood for women in Nepal is linked to marriage, fertility and acceptance into her husband׳s family (Bennett, 1983, Cameron, 1998, Winkvist and Akhtar, 2000). Furthermore, marriage is the primary means of accessing financial support for most Nepali women. The disabled women we interviewed wanted and expected to have children after marriage, and their families also shared this expectation. As in many parts of South Asia, being a single woman, having a child out of wedlock, or having a childless marriage is stigmatised in Nepal (Weiss, 1999, Nahar, 2010, Jordal et al., 2013).

Research suggests that most disabled women in Nepal are single (UNESCAP, 1995) and they may face increased stigma, being disabled, single and childless. Because the women in our study had confirmed their identity through marriage and motherhood, their impairment may have disabled them to a lesser extent. This may also help to explain the high level of triangulation between issues affecting disabled and non-disabled women.

## Conclusion

Surprisingly, we found that there was little difference in the reasons for home delivery among disabled and non-disabled women. Married disabled women considered pregnancy and childbirth to be normal, but health workers felt unprepared to meet the maternal health needs of disabled women. This suggests that health workers could benefit from training integrated into existing curricula. Although Nepal is making good progress towards meeting the maternal health Millennium Development Goals; issues of quality, cost and lack of family support still prevent women from having an institutional delivery. These issues are as pertinent for disabled women as they are for their non-disabled peers. There is a need to monitor progress of interventions to encourage institutional delivery through the use of disaggregated data, to check that disabled women are benefiting equally in efforts to improve maternal health. Our study also illustrates the importance of consulting disabled women themselves to obtain a more accurate understanding of their experience of maternal health care seeking.

## Conflict of interest statement

The authors declare that they have no conflict of interest.
